# Pur-alpha functionally interacts with FUS carrying ALS-associated mutations

**DOI:** 10.1038/cddis.2015.295

**Published:** 2015-10-22

**Authors:** M Di Salvio, V Piccinni, V Gerbino, F Mantoni, S Camerini, J Lenzi, A Rosa, L Chellini, F Loreni, M T Carrì, I Bozzoni, M Cozzolino, G Cestra

**Affiliations:** 1 IBPM, Istituto di Biologia e Patologia Molecolari, CNR, Rome, Italy; 2 University of Rome 'Sapienza', Department of Biology and Biotechnology, Rome, Italy; 3 Laboratory of Neurochemistry, Fondazione Santa Lucia, IRCCS, Rome, Italy; 4 Department of Cell Biology and Neurosciences, Istituto Superiore di Sanità, Rome, Italy; 5 Center for Life Nano Science@Sapienza, Istituto Italiano di Tecnologia, Rome, Italy; 6 Department of Biology, University of Rome 'Tor Vergata', Rome, Italy; 7 IFT, Istituto di Farmacologia Traslazionale, CNR, Rome, Italy

## Abstract

Amyotrophic lateral sclerosis (ALS) is a fatal neurodegenerative disorder due to motor neuron loss. Fused in sarcoma (FUS) protein carrying ALS-associated mutations localizes to stress granules and causes their coalescence into larger aggregates. Here we show that Pur-alpha physically interacts with mutated FUS in an RNA-dependent manner. Pur-alpha colocalizes with FUS carrying mutations in stress granules of motoneuronal cells differentiated from induced pluripotent stem cells and that are derived from ALS patients. We observe that both Pur-alpha and mutated FUS upregulate phosphorylation of the translation initiation factor eukaryotic translation initiation factor 2 alpha and consistently inhibit global protein synthesis. *In vivo* expression of Pur-alpha in different Drosophila tissues significatively exacerbates the neurodegeneration caused by mutated FUS. Conversely, the downregulation of Pur-alpha in neurons expressing mutated FUS significatively improves fly climbing activity. All these findings suggest that Pur-alpha, through the control of mRNA translation, might be involved in the pathogenesis of ALS associated with the mutation of FUS, and that an alteration of protein synthesis may be directly implicated in the disease. Finally, *in vivo* RNAi-mediated ablation of Pur-alpha produced locomotion defects in *Drosophila,* indicating a pivotal role for this protein in the motoneuronal function.

Amyotrophic lateral sclerosis (ALS) is a severe neurodegenerative disorder caused by motor neuron loss in the brain and spinal cord.^1^ Several gene mutations are causative of the familiar form of the disease and the corresponding mutant proteins often mislocalize and aggregate in the cytoplasm. This is the case of fused in sarcoma/translocated in liposarcoma (FUS/TLS or FUS).^2, 3^ FUS is a nuclear DNA/RNA-binding protein that contains nuclear import and export signals and regulates transcription, splicing, and mRNA metabolism.^1^ In familiar ALS FUS mutations often map in the C-terminal proline/tyrosine-nuclear localization signal (PY-NLS).^4, 5^ While wild-type FUS localizes in the nucleus, mutant protein often localizes in the cytoplasm, where eventually coalesces into stress granule (SG) aggregates.^6^ Mutation in the PY-NLS motif, although resulting in the abnormal cytosolic localization of FUS, may not be sufficient for its recruitment in SGs. Therefore, we hypothesize that alterations of a protein–protein interaction network around the C-terminus of FUS may account for its localization in SGs, affecting ALS pathogenesis. By affinity purification experiments from rat total brain extract, we identified Pur-alpha as a protein that specifically binds to FUS C-terminal fragment. Pur-alpha is a highly conserved protein, which interacts in a sequence-specific manner with single-stranded DNA and RNA.^7^ It is involved in targeting mRNA to neuronal dendrites,^8^ in DNA replication, DNA repair, and gene transcription and it associates to the TAR RNA element of HIV-1.^9, 10, 11^ Pur-alpha knockout mice die within 4 weeks of major neurological disorders.^12^ Very interestingly, Pur-alpha was recently demonstrated to bind to GGGGCC expanded repeats of C9orf72 gene, which represents the most frequent mutation associated with familiar ALS.^13^ In a *Drosophila* model of neurodegeneration caused by GGGGCC repeats expression, Pur-alpha ameliorates the phenotype.^14^ Here we provide new evidence for a role of Pur-alpha in the regulation of translation and SG formation and we suggest that it may be involved in the pathogenesis of FUS-mediated ALS.

## Results

### Identification of Pur-alpha as an FUS-binding protein

To identify the protein–protein interaction network involving the last 17 residues of FUS we generated glutathione *S*-transferase (GST) fusions of the C-terminus of FUS to use as bait in affinity purification experiments. We subcloned the human cDNA encoding the last 51 residues of wild-type FUS (FUS_Ct_WT), which encompasses a small fragment of the last RNA-binding domain, to leave enough space between the GST tag and the C-terminal motif. Then we mutagenized FUS_Ct_WT construct introducing four ALS-associated mutations (R521G, R522G, R524S, and P525L) and we generated a construct named hereafter Multiple Mutant, MM (FUS_Ct_MM). We used GST-tagged FUS_Ct_WT and FUS_Ct_MM, and GST on its own in affinity purification experiments from brain Triton X-100 extracts. By nanoflow reversed-phase liquid chromatography tandem mass spectrometry we identified several FUS-binding partners. We found interactors that bind FUSWT fragment, but less efficiently FUSMM, and proteins that showed an increased affinity for mutated FUS.

Although we utilized only a small fragment of FUS of 51 amino acids as bait, we were able to identify 24 putative binding partners that differentially interact with one or another of the two forms of the C-terminal peptide. Among all these partners PABP and NonA, involved in SG and paraspeckle formation respectively,^2, 15^ were already known as FUS-binding proteins. Of these 24 partners we found 7 RNA-binding proteins, 5 mRNA translation regulators, 4 DNA-binding proteins involved in transcriptional control, 4 proteins involved in microtubule assembly, and 4 interactors either with an unknown function or involved in cell signaling and mitochondrial regulatory activity.

**Figure 1:**
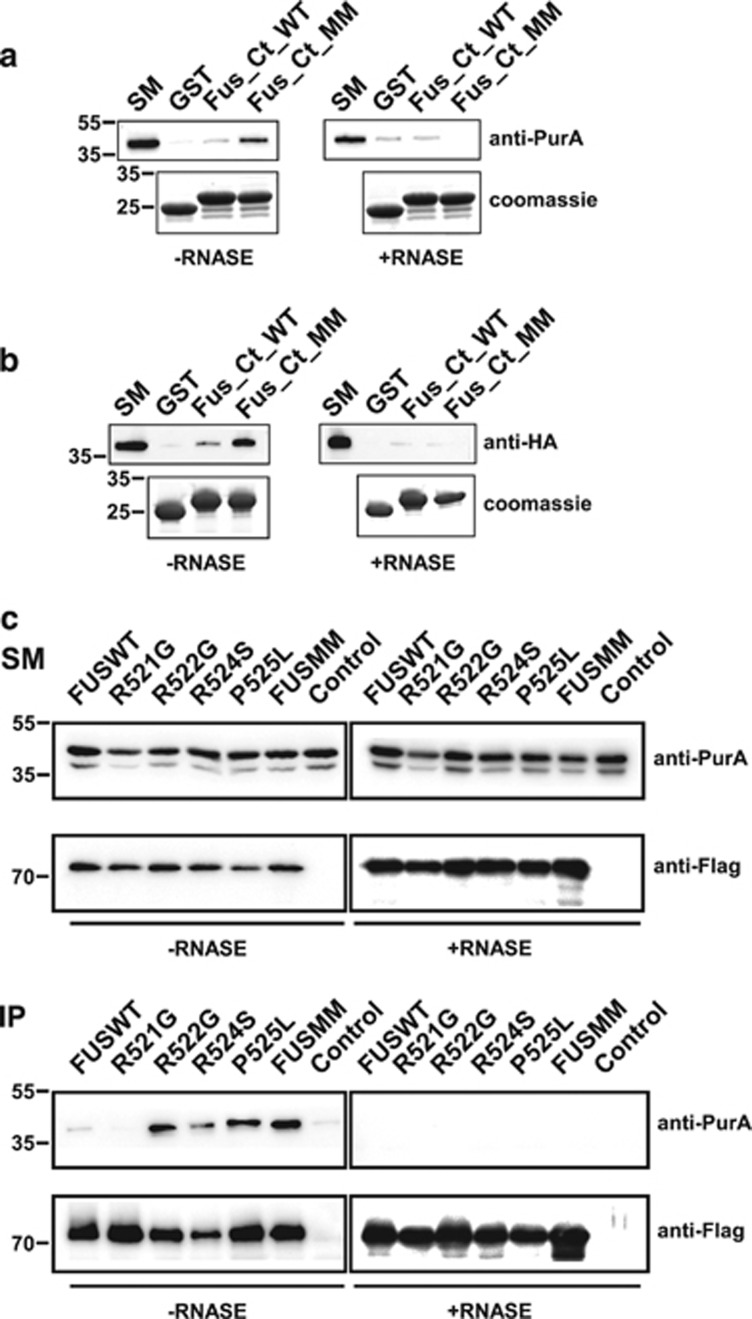
FUS/Pur-alpha physical interaction. (**a**) GST, GST-fused C-terminal region of wild-type FUS (FUS_Ct_WT), and GST C-terminal domain of multimutated FUS (FUS_Ct_MM) were used as baits in affinity purification experiments from a rat brain Triton X-100 extract, in the presence or absence of RNAse. Affinity-purified material retained by the GST fusion proteins was resolved by SDS-PAGE and processed by western blotting with anti-Pur-alpha antibody (top). The same volume of eluted material analyzed by western blotting was separated on a different SDS-PAGE and stained with Coomassie blue to verify equal loading of the different GST fusion baits (bottom). 1 : 500 of total brain extract and 1:50 of proteins retained from each column were loaded on the gel. SM, starting material. (**b**) Interaction between the same GST fusion proteins utilized in (**a**) and *in vitro*-translated HA-tagged Pur-alpha was tested by pull-down in the presence or absence of RNAse. HA Pur-alpha bound to the GST fusion proteins was resolved by SDS-PAGE and analyzed by western blotting with anti-HA antibody. GST fusion proteins used in the pull-down assay were resolved by SDS-PAGE and stained with Coomassie blue (bottom). 1 : 50 of reticulocyte extract exploited in the pull down and 1:3 of proteins retained from each column were loaded on the gel. (**c**) Protein extracts from HeLa cells expressing HA-Pur-alpha on its own (Control), or together with FUSWT, FUS carrying single mutations (R521G, R522G, R524S, or P525L), or FUSMM were incubated with or without RNAse and immunoprecipitated with anti-Flag antibody. Retained proteins were separated by SDS-PAGE and analyzed by western blotting with anti-HA and anti-Flag antibodies. 1 : 50 of total cell extract utilized for each immunoprecipitation and 1 : 3 of bound proteins were loaded on the gel. SM, starting material; IP, immunoprecipitate

Among interactors that bind strongly mutated FUS motif we directed our attention on Pur-alpha. To characterize the interaction between FUS C-terminal region and Pur-alpha, GST fragments were incubated with a Triton X-100 extract from rat brain, with or without RNAse. As shown in Figure 1a (left panel), Pur-alpha binds preferentially FUSMM and to a minor extent FUSWT. The interaction is almost completely abolished by RNAse treatment (right panel). FUS/Pur-alpha interaction was also confirmed in a pull-down assay in which GST FUS fragments were incubated with *in vitro-*translated hemagglutin (HA)-tagged Pur-alpha (Figure 1b).

To isolate a stable complex containing both full-length proteins, and to analyze binding activity of the single mutations of FUS to Pur-alpha, we immunoprecipitated FUS from HeLa cells co-expressing each Flag-FUS mutant with HA-Pur-alpha. As indicated in Figure 1c, while R522G, P525L, and FUSMM show a robust interaction with Pur-alpha, FUSWT, R521G, and R524S bind very weakly.

### Pur-alpha colocalizes with FUS protein carrying ALS-associated mutations

**Figure 2:**
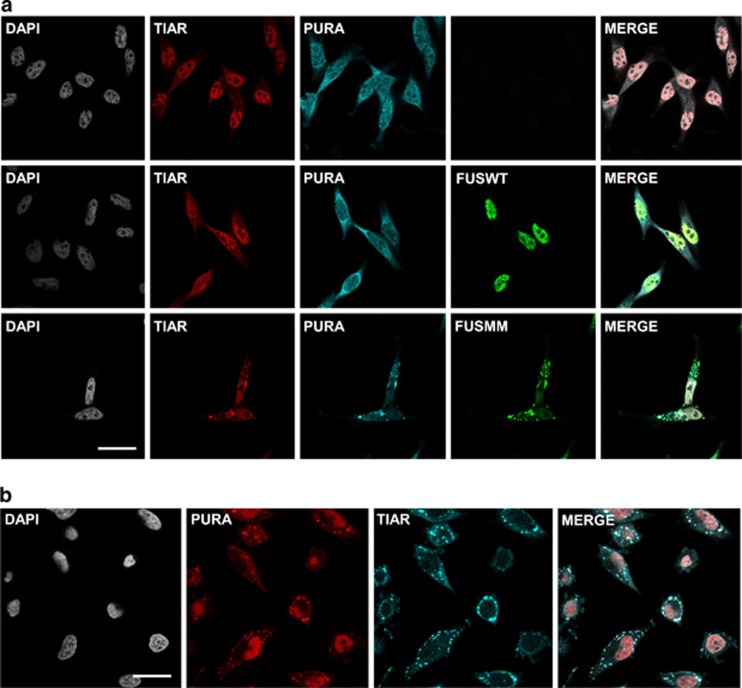
Immunofluorescence staining of FUS and Pur-alpha proteins. (**a**) Effect of FUSMM expression on endogenous Pur-alpha localization. Untransfected HeLa cells or cells expressing either Flag-FUSWT or Flag-FUSMM were labeled by immunofluorescence with anti-Pur-alpha, anti-Flag, and with antibodies directed against the stress granule marker TIAR (bars=10 *μ*m). (**b**) Untransfected HeLa cells were treated for 30 min with 1 mM of sodium arsenite and stained by immunofluorescence with anti-Pur-alpha and anti-TIAR antibodies (bars=20 *μ*m)

We transfected HeLa and NSC-34 (data not shown) cells with Flag-FUSWT or Flag-FUSMM and we visualized, by immunofluorescence, endogenous Pur-alpha in transfected and untransfected cells (Figure 2a). Since FUS mutants often relocalize in the cytoplasm where it coalesce into SGs, we studied FUS and Pur-alpha localization compared with the SG marker TIAR (TIA-1 related protein). Pur-alpha, which is distributed in both nuclear and cytoplasmic compartments, showed a partial co-localization with FUSWT, which localizes exclusively in the nucleus. In cells overexpressing FUSMM, which forms large SG aggregates, Pur-alpha strongly colocalizes with FUSMM in cytoplasmic inclusions (Figure 2a). Pur-alpha behaves as an SG protein and it strongly relocalizes together with TIAR in cells treated with the oxidative stress-producing agent sodium arsenite (Figure 2b).

**Figure 3:**
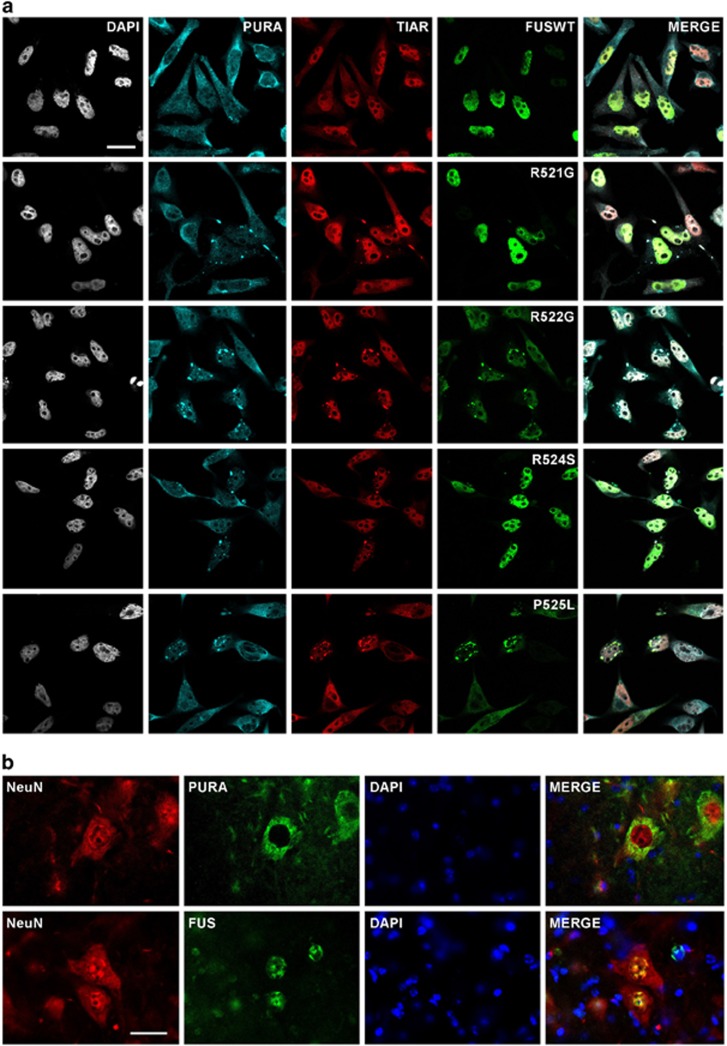
Co-localization of endogenous Pur-alpha with FUS proteins carrying single ALS-associated mutations. (**a**) HeLa cells transfected with plasmids encoding Flag-FUSWT or FUS carrying the indicated single C-terminal mutations were stained by immunofluorescence with anti-Pur-alpha, anti-Flag, and anti-TIAR antibodies (bars=10 *μ*m). (**b**) Localization of endogenous FUS and Pur-alpha in mouse spinal cord as demonstrated by the counterstain with antibodies directed against the neuronal marker NeuN (bars=20 *μ*m)

To study the localization of Pur-alpha in cells expressing single mutation of FUS we performed immunofluorescence labeling of HeLa cells transfected with plasmids encoding each Flag-FUS mutant. As shown in Figure 3, FUSWT, R521G, and R524S, which are distributed almost exclusively in the nucleus, co-localize with the nuclear pool of Pur-alpha. Differently, R522G, P525L, and FUSMM, which strongly relocalize in cytoplasmic aggregates, show a prominent co-localization with endogenous Pur-alpha. Interestingly, in a significant population of cells expressing R521G and R524S mutants, which generate only very small pool of cytoplasmic protein and rarely form visible aggregates, Pur-alpha and TIAR relocalize in SGs (Figure 3a). No SGs are observed in cells expressing FUSWT. Thus Pur-alpha relocalizes in SGs and strongly colocalizes with those FUS mutants that prominently aggregate in the cytoplasm.

To characterize FUS aggregations in HeLa cells we analyzed the endogenous localization of TAR DNA-binding protein-43 (TDP-43) in cells expressing different FUS mutants. As shown in Supplementary Figure S1 TDP-43 colocalizes with FUSWT in the nucleus. In cells with small cytoplasmic aggregations of FUS R522G or large inclusions of P525L, TDP-43 does not show any major relocalization in the cytoplasm. Thus, we do not observe any significant co-localization of TDP-43 with FUS aggregates.

### FUS and Pur-alpha are coexpressed in mouse spinal cord neurons

To investigate whether Pur-alpha is expressed in cell types more relevant for ALS, we studied its localization in mouse spinal cord sections. We stained by immunofluorescence mouse spinal cord cells with either anti-Pur-alpha or anti-FUS antibodies together with an antibody against Neuronal Nuclei protein NeuN, used as a neuronal marker (Figure 3b). Both Pur-alpha and FUS are consistently expressed in neuronal cells from the spinal cord. Furthermore, wild-type FUS and Pur-alpha do not show major co-localization in neurons: while FUS is enriched in the nucleus, Pur-alpha accumulates in the cytoplasm.

### Pur-alpha colocalizes with FUS mutant proteins in motoneurons differentiated from induced pluripotent stem cell lines

**Figure 4:**
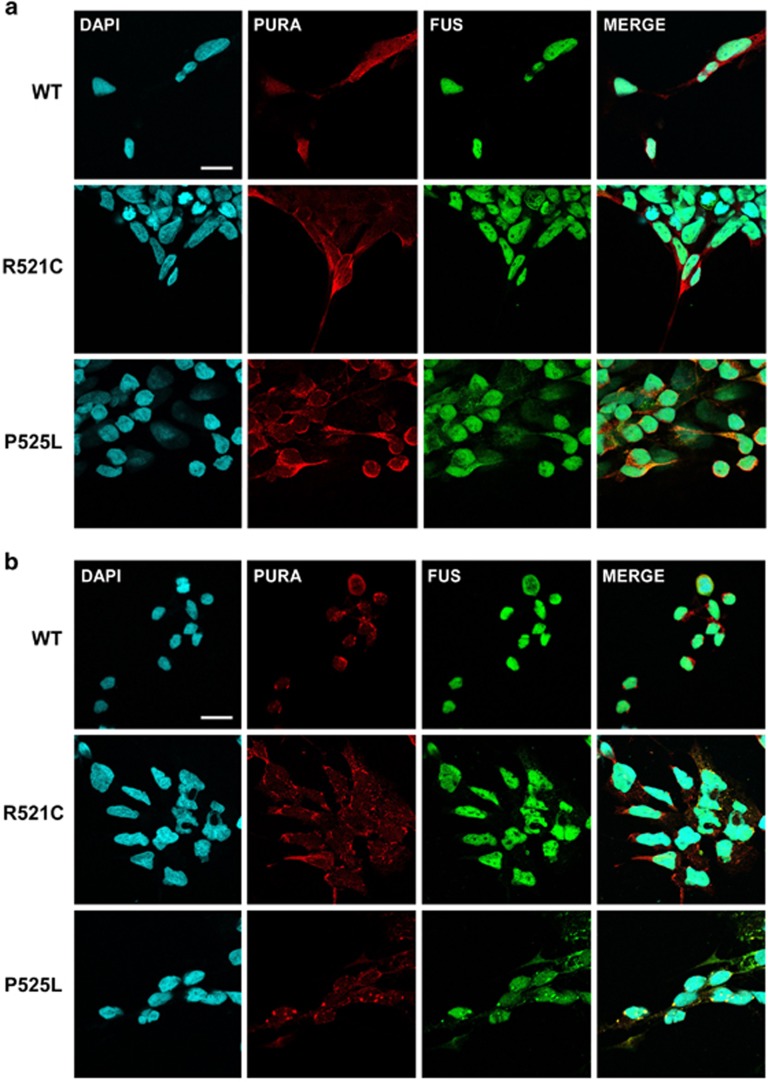
Co-localization of Pur-alpha with mutated FUS in motoneurons differentiated from IPSCs. (**a**) Untreated motoneurons differentiated from IPSC cells, which derived from ALS patients or from healthy donor (WT), were labeled by immunofluorescence with anti-Pur-alpha and anti-FUS antibodies (bars=10 *μ*m). (**b**) Same motoneurons shown in (**a**) were treated with 0.5 mM of sodium arsenite for 90 min to induce the formation of stress granules and stained by immunofluorescence with anti-Pur-alpha and anti-FUS antibodies (bars=10 *μ*m)

To confirm the functional relationship of Pur-alpha with FUS in a more relevant model system for the disease, we studied the localization of Pur-alpha in induced pluripotent stem cell lines (iPSCs), differentiated into ventral spinal cord populations containing motoneurons. In the experimental conditions used, a relevant fraction of cells differentiates in motoneurons, as assessed by immunofluorescence staining of the motor neuronal markers islet1/2 (Supplementary Figure S2). As these cell cultures can be used to model ALS *in vitro*,^16^ we analyzed the localization of Pur-alpha in motoneurons differentiated from three different genetic backgrounds (Figures 4a and b): wild type, R521C heterozygous, or P525L homozygous FUS mutations. Under standard conditions, wild type and mutant proteins are primarily accumulated in the nucleus, with the only exception of FUS-P525L, which shows a significant cytoplasmic pool (Figure 4a). However, under these conditions, no aggregates of FUS are observed (Figure 4a). In cells treated with the oxidative stress-producing agent sodium arsenite, we observe a major relocalization of Pur-alpha in SGs (Figure 4b). While localization of wild-type FUS is not affected by sodium arsenite, in the P525L mutant the cytoplasmic pool of FUS strongly relocalizes in SGs, together with Pur-alpha. Although to a lesser extent, FUS relocalization is also observed in a subset of R521C mutant cells (Figure 4b). Thus Pur-alpha strongly colocalizes with cytoplasmic aggregates of mutated FUS.

### Pur-alpha and mutated FUS promote eukaryotic translation initiation factor 2 alpha phosphorylation and abolish incorporation of puromycin

**Figure 5:**
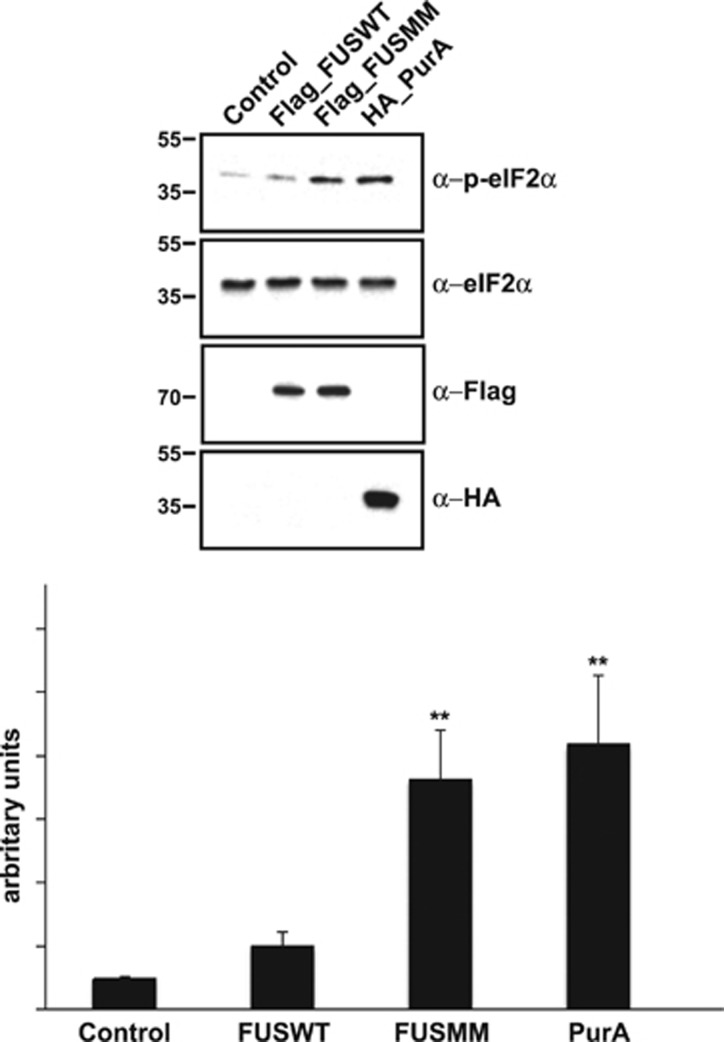
Effect of Pur-alpha and FUSMM expression on eIF2-alpha phosphorylation of HEK293-starved cells were transfected with HA-Pur-alpha, Flag-FUSWT, or Flag-FUSMM. eIF2-alpha phosphorylation was evaluated by western blotting with anti-p-eIF2-alpha antibody. In starved HEK293 cells the expression of HA-Pur-alpha determines a strong induction of eIF2-alpha phosphorylation as well as Flag-FUSMM, if compared with cells expressing Flag-FUSWT or untransfected cells. The lower panel shows band quantification generated with ImageJ analysis package. Data are presented as the ratio of phospho-eIF2-alpha to total eIF2-alpha signal and are expressed in arbitrary units (AU). Values are reported as mean±S.D.; *n*=3. Statistical significance was evaluated with Student's *t*-test (** indicate values significantly different from controls with *P*-value <0.001)

**Figure 6:**
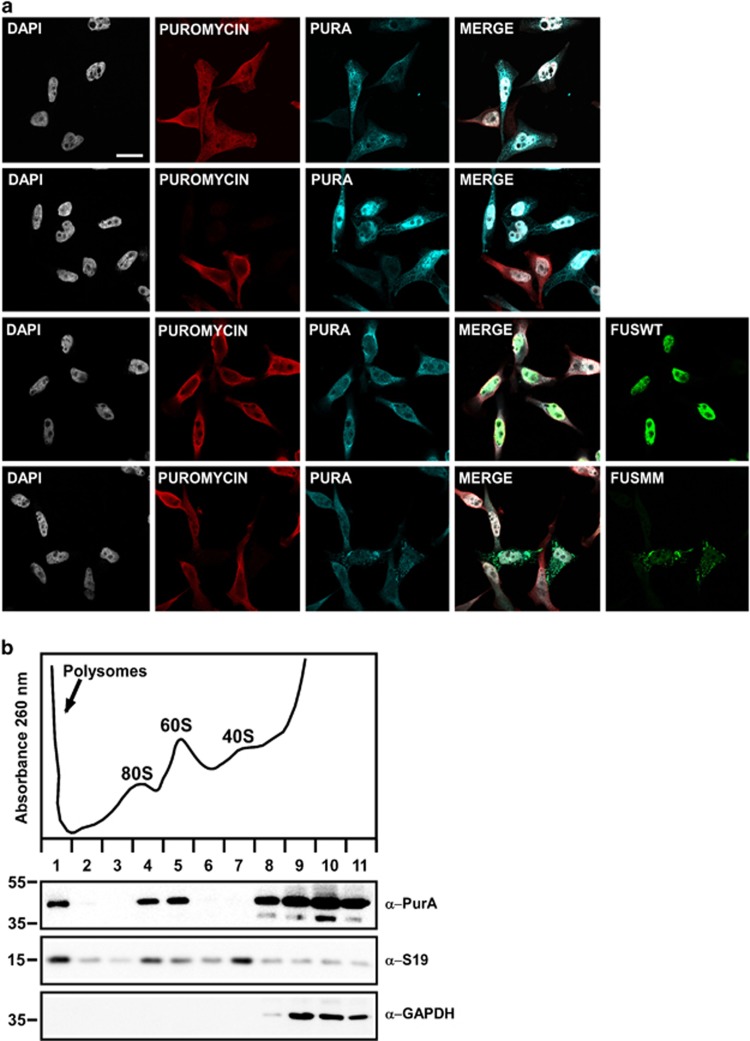
Effect of Pur-alpha and FUSMM expression on protein synthesis. (**a**) Untransfected HeLa cells or cells transfected with HA-Pur-alpha, Flag-FUSWT, or Flag-FUSMM were treated with puromycin. Untransfected cells are shown in the first row, while cells transfected with HA-Pur-alpha are in the second row. Cells with higher expression of Pur-alpha (transfected cells) show no incorporation of puromycin, compared with untransfected cells of the same field. Puromycin incorporation was detected by immunofluorescence with anti-puromycin antibody. Cells were also labeled by immunofluorescence with anti-Pur-alpha and anti-Flag antibodies (bars=10 *μ*m). (**b**) Cytoplasmic extract from HEK293 cells was separated by ultracentrifugation on a linear sucrose gradient. Eleven fractions were collected monitoring the absorbance at 260 nm and the pellet containing polyribosomes was pooled to the first fraction. The upper panel shows the absorbance profile with the position of ribosomal subunits and monomer indicated. Proteins purified from the fractions were analyzed by western blot with the indicated antibodies

Untranslated mRNAs localize to SGs in cells exposed to environmental stress.^17, 18^ Phosphorylation of eukaryotic translation initiation factor 2 alpha (eIF2-alpha), inhibiting translational initiation, allows elongating ribosomes to run off mRNAs, which accumulate in SGs. eIF2-alpha phosphorylation is commonly used as a biochemical marker of SG formation. Thus we investigated the involvement of Pur-alpha in the regulation of SG formation analyzing the degree of eIF2-alpha phosphorylation in cells expressing Pur-alpha and FUS. Cells transfected with FUSMM or with Pur-alpha show a significant increase of eIF2-alpha phosphorylation compared with untransfected cells or cells expressing FUSWT (Figure 5). To confirm the role of Pur-alpha and FUSMM in the regulation of mRNA translation we measured rates of global protein synthesis, through a puromycin incorporation assay, in cells expressing both proteins. Puromycin works as analogous of aminoacyl-tRNA and causes premature release of polypeptide chains with formation of polypeptidyl-puromycin derivatives. Thus the incorporation rate of puromycin directly reflects protein synthesis activity.^19, 20^ We treated transfected and untransfected HeLa cells with puromycin and we followed its incorporation by immunofluorescence using an anti-puromycin antibody. As shown in Figure 6a, untransfected cells and cells expressing FUSWT show a significant incorporation of puromycin, while in cells expressing either Pur-alpha or FUSMM the incorporation of puromycin is almost absent. Then we measured puromycin incorporation in cells expressing each mutant of FUS. As shown in Supplementary Figure S3, we observe a prominent reduction of puromycin incorporation in all cells with a minimal pool of aggregated FUS in the cytoplasm. While only a small number of cells expressing R521G and R524S show cytoplasmic aggregates of FUS, which nevertheless correlate with a prominent reduction of protein synthesis, the vast majority of cells expressing R522G and P525L shows visible aggregates associated with a robust reduction of puromycin incorporation. Thus, either the expression of Pur-alpha or of any FUS mutant that forms cytoplasmic aggregates strongly reduces the rate of global protein synthesis.

### Pur-alpha associates with ribosomes

To gain more insight into the role of Pur-alpha in the regulation of translation we evaluated its association with ribosomal particles. Cytoplasmic extracts from HEK293 were separated on a linear sucrose gradient and proteins isolated from the different fractions were analyzed by western blot. As shown in Figure 6b a significant pool of Pur-alpha cosediments together with 60S ribosomal subunits, with monomeric 80S ribosomal particles and with polyribosomes, suggesting a physical association of Pur-alpha with ribosomes.

### RNAi-mediated downregulation of Pur-alpha in *Drosophila* alters fly locomotion

**Figure 7:**
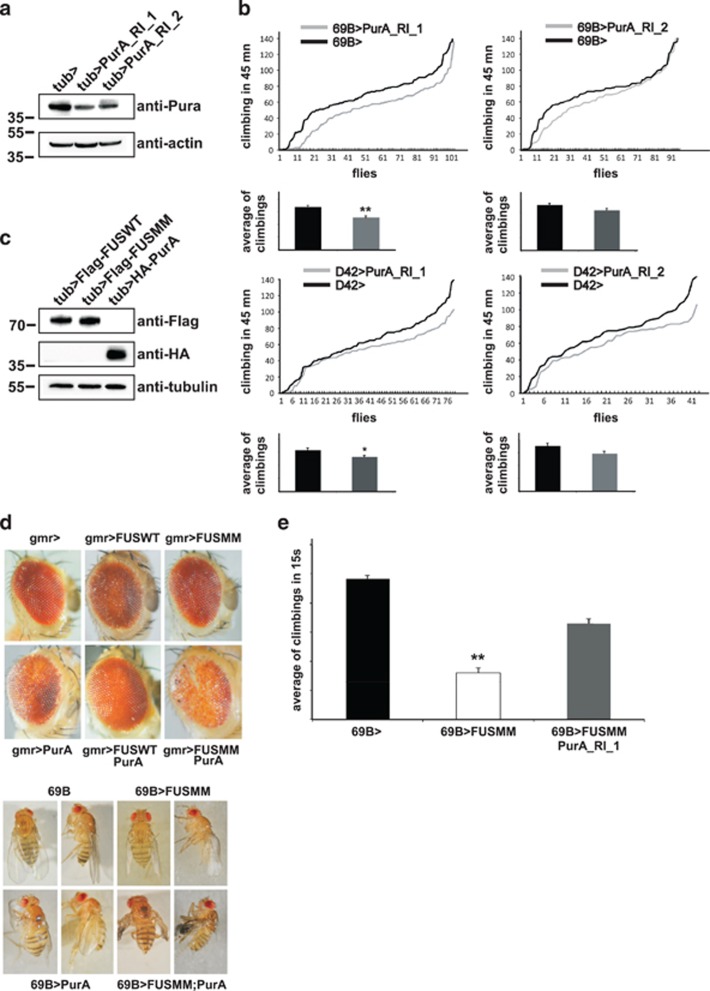
*In vivo* role of Pur-alpha in *Drosophila melanogaster*. (**a**) RNAi mediated downregulation of Pur-alpha in all *Drosophila* tissues by the expression of Pur_alpha_RNAi_1 and Pur_alpha_RNAi_2 RNAi under control of the ubiquitous driver tubulin-GAL4. Total extracts from RNAi expressing flies and control animals were separated by SDS-PAGE and the extent of Pur-alpha downregulation was evaluated by western blotting with an anti-Pur-alpha antibody. (**b**) The same RNAi fly lines of (**a**) were crossed at 29 °C with the pan neuronal driver 69B (upper panels) and with the motoneuron driver D42 (lower panels). Climbing performance of each offspring is represented by plotting the total number of climbing events for each fly of the group. Numbers of climbing events for all flies of the group were ascending ordered and plotted. Statistical significance was evaluated with Student's *t*-test (** high statistical significance, *P*-value <0.001; * statistical significance, *P*-value <0.05) and the averages of climbing events in each population, with corresponding standard errors, are shown. (**c**) Expression level of FUSWT, FUSMM, and Pur-alpha mammalian proteins in fly eyes. Heads from flies expressing the transgenes under GMR were separated and homogenized. Protein extracts were separated on SDS-PAGE and the expression of each transgene was evaluated by western blotting. (**d**) Genetic interaction of FUSMM and Pur-alpha in *Drosophila* eye. Eyes of flies expressing the mammalian genes under control of GMR Gal4 are shown. FUSWT and Pur-alpha induces respectively very mild and mild eye degeneration, while expressing FUSMM does not determine any visible phenotype. A simultaneous expression of both FUSMM and Pur-alpha causes strong eye degeneration. Pictures of flies expressing the transgenes under the pan neuronal driver 69B are shown. FUSWT expression causes early fly lethality (not shown), while FUSMM induces an alteration of wing extension; similar unextended wings are observed in flies expressing Pur-alpha. Expression of both FUSMM and Pur-alpha generates a more severe alteration of wing morphology. (**e**) Fly lines expressing FUSMM on its own, or combined with Pur_alpha_RNAi_1, were crossed with 69B-GAL4 pan neuronal driver, at 25 °C. A negative geotaxis assay was performed to measure climbing activity of flies with different genotypes. Averages of climbing evens are shown together with corresponding standard errors. Downregulation of Pur-alpha in neurons expressing FUSMM significatively improves fly climbing activity. Statistical significance was evaluated with Student's *t*-test (values significantly different from relative controls are indicated with two asterisk; *P*<0.001)

To gather functional evidence on the *in vivo* role of Pur-alpha we examined locomotion activity of *Drosophila melanogaster* flies in which Pur-alpha expression was specifically inactivated by RNAi in neurons and motoneurons. In Figure 7a is shown the extent of RNAi-mediated reduction of Pur-alpha expression in two independent fly lines (Pur-alpha_RI_1; Pur-alpha_RI_2), analyzed by western blotting. The same lines were crossed with the pan neuronal promoter 69B and the offspring, grown at 29 °C, was studied using a *Drosophila* activity monitoring system. Flies of both lines show a reduction of climbing activity, which reaches statistical significance for Pur_alpha_RNAi_1 flies (Figure 7b, upper panels). Correspondingly, flies grown at 29 °C and expressing the same RNAi constructs under control of the motoneuronal promoter D42 show climbing defects (Figure 7b, lower panels), and again the impairment of Pur_alpha_RNAi_1 flies reaches statistical significance. Consistently, Pur_alpha_RNAi_1 produces a more efficient downregulation of Pur-alpha expression compared with Pur-alpha_RI_2 (Figure 7a).

### Coexpression of FUSMM and Pur-alpha exacerbates degeneration in *Drosophila*

To characterize the functional relationship between FUS and Pur-alpha in the *in vivo Drosophila* model system we generated flies that coexpress mammalian FUS and Pur-alpha proteins under UAS promoter. Utilizing the phiC31 integrase system we produced two different transgenic fly lines in which FUSWT and FUSMM were inserted in the same genomic site, assuring the same expression level (Figure 7c). With further crosses we generated flies carrying FUSWT and Pur-alpha transgenes, and flies carrying FUSMM and Pur-alpha. Using the glass multimer reporter driver line we expressed these mammalian genes in fly eye at 25 °C. Eye degeneration was observed in flies expressing FUSWT or Pur-alpha (Figure 7d, upper panel), while any alteration of fly eyes was observed in flies expressing FUSMM on its own (Figure 7d, upper panel). Combining the expression of FUSWT and Pur-alpha we do not observe any relevant modification of the eye phenotype produced by each single transgene. Conversely, the coexpression of Pur-alpha and FUSMM causes stronger eye degeneration than each single expression (Figure 7d, upper panel). Parallel results were obtained expressing these proteins under the pan neuronal driver 69B at 25 °C (Figure 7d, lower panel): while FUSWT expression causes early fly lethality, expressing FUSMM produces visible wing alterations, very similar to the phenotype observed in flies expressing Pur-alpha on its own. Again, combining the expression of both FUSMM and Pur-alpha we obtained more severe alterations and a strong inhibition of wing extension (Figure 7d, lower panel). Thus, we addressed whether the alterations due to the expression of FUSMM, Pur-alpha, or both, correlated with an increase in neuronal cell death. We generated *Drosophila* larvae expressing these proteins in all neurons, under control of the ELAV (embryonic lethal abnormal vision)-Gal4 (galactose-responsive transcription factor) driver, and whole mount brains from these animals were stained with anti-active Caspase-3 antibodies (Supplementary Figure S4). As shown in Supplementary Figure S4, we observe a consistent labeling of active Caspase-3, a well-known marker of cell death, in neuronal cells expressing FUSMM, Pur-alpha, or both, while almost no staining is visualized in brains expressing ELAV-Gal4 on its own.

### Downregulation of endogenous Pur-alpha in flies expressing FUSMM ameliorates climbing activity

Since coexpression of both FUSMM and Pur-alpha produces more severe phenotypes compared with the expression of each single protein, we investigated the effect of Pur-alpha downregulation in flies expressing FUSMM. We generated flies expressing FUSMM on its own, or combined to Pur_alpha_RNAi_1 construct, under the pan neuronal driver 69B-GAL4. Climbing activity of flies grown at 25 °C was analyzed in a negative geotaxis assay (Figure 7e). Flies expressing FUSMM and Pur_alpha_RNAi_1 constructs show a significant improvement in climbing activity compared with flies expressing FUSMM.

## Discussion

Our results provide new evidence for a role of Pur-alpha in the regulation of translation and SGs and, given its preferential binding to ALS-linked FUS mutant proteins, we suggest that it may be involved in the pathogenesis of FUS-mediated ALS.

We have shown that Pur-alpha binds specifically, *in vitro* and *in vivo*, to the C-terminal region of FUS carrying ALS-associated mutations, in an RNA-dependent manner (Figure 1). We have observed that FUS and Pur-alpha are expressed in spinal cord motoneurons (Figure 3b), and that mutated FUS colocalizes with Pur-alpha in SGs (Figures 2a and 3a). More interestingly, Pur-alpha strongly colocalizes with mutated FUS in motoneurons differentiated from IPSCs (Figure 4). According to a close connection between FUS and Pur-alpha, we observe that either Pur-alpha or FUSMM expression causes the upregulation of eIF2-alpha phosphorylation (Figure 5), a signature of translational inhibition. In a puromycin incorporation assay, used to quantify the global rate of protein synthesis, we confirmed that their expression arrests mRNA translation (Figure 6a and Supplementary Figure S3). Consistently, through the sedimentation of cytoplasmic extracts on a sucrose gradient we also observe a prominent pool of Pur-alpha protein associated with free 60 S ribosomal subunits, monomeric ribosomal particles, and polyribosomes (Figure 6b). A role of Pur-alpha in translation is consistent with its observed association with RAR-alpha and fragile X mental retardation protein in ribonucleoprotein complexes containing translationally silenced mRNA,^21^ or with its inhibitory activity in an *in vitro* assay of protein translation, through the interaction with 18 S homologous ribosomal RNA.^22^ Although several pieces of evidence point to a direct role of Pur-alpha in the regulation protein synthesis, we cannot rule out that it may also affect mRNA translation indirectly through the potentiation of the stress response pathway.

Pur-alpha expression in *Drosophila* tissues (either eyes or wings) exacerbates FUSMM phenotypes (Figure 7d), while its downregulation ameliorates locomotion defects caused by FUSMM expression (Figure 7e). Because both FUSMM and Pur-alpha upregulate eIF2-alpha phosphorylation (Figure 5) and inhibit puromycin incorporation (Figure 6a and Supplementary Figure S3), we propose that FUSMM and Pur-alpha affect parallel pathways converging on the inhibition of protein translation. Our results from *Drosophila* suggest that Pur-alpha may contribute to the neurodegeneration caused by the cytoplasmic function of FUS mutant proteins, and imply that particular human genetic backgrounds, in which Pur-alpha expression is downregulated, may play protective effects on the pathological role of FUS. Thus we propose that the inhibition of translation might be involved in FUS-mediated ALS. This is in good agreement with what was recently observed in a TDP-43 *Drosophila* model for ALS, in which eIF2-alpha phosphorylation was increased by TDP-43 toxicity.^23^

Our results also indicate that the neurotoxicity mediated by wild type or mutated FUS expression may occur through different pathways. FUSWT-mediated neurodegeneration in the eye is not affected by Pur-alpha expression, and FUSWT does not substantially upregulate eIF2-alpha phosphorylation in cultured cells. Conversely, FUSMM enhances Pur-alpha neurodegeneration and promotes eIF2-alpha phosphorylation. In addition, we observed a different degree of alteration caused by the *in vivo* expression of either FUSWT or FUSMM, with a stronger phenotype often associated with FUSWT overexpression. While the expression of FUSWT under control of the pan neuronal driver 69B causes early fly lethality, FUSMM expression, under the same conditions, induces only a wing phenotype (Figure 7d). This is even more relevant as we compared effects caused by transgenes inserted in the same genomic site and that produce very similar levels of expression (Figure 7c). Indeed, these results are in agreement with several reports in which a severe ALS was caused by the upregulation of wild-type FUS expression.^24, 25, 26^ Furthermore, we observe different effects of wild type and mutated FUS on SGs. Cells expressing R521G and R524S mutants, although show very small pool of cytoplasmic protein and undetectable protein aggregates, induce a relevant relocalization of Pur-alpha and TIAR in SGs (Figure 3), while no relocalization is observed in cells expressing FUSWT. Thus a minimal pool of cytoplasmic mutated FUS may be sufficient to form SGs.

Finally, we also observed as the ablation of Pur-alpha by RNAi in neurons and motor neurons affects, to a different extent, locomotion activity of flies generating ALS-like phenotypes (Figure 7b). Data are consistent with the phenotype of Pur-alpha knockout mice that display a continuous tremor starting at the age of 2 weeks, associated with other major neurological disorders.^12^

In conclusion, we unveil a specific physical and genetic interaction between Pur-alpha and FUS carrying ALS causative mutations and a novel functional role of Pur-alpha in the regulation of SGs and protein synthesis. Thus, we suggest that Pur-alpha may contribute to the recruitment of mutated FUS proteins in SGs, and potentiating their translational inhibitory effect, it may be actively involved in FUS toxicity.

## Materials and Methods

### Drosophila methodologies

Transgenic flies expressing Flag-FUSWT and Flag-FUSMM were generated by the Bestgene Service Company using the phiC31 integrase system to insert each pUAST-attB vector in the second chromosome (strain 24481). A HA-Pur-alpha transgenic fly line was produced by phiC31 integrase-mediated insertion of the pUAST-attB vector in the strain 24749, on the third chromosome. RNAi fly lines targeting Pur-alpha gene expression (purA_RI_1, v101363; purA_RI_2, v48249) were obtained from Vienna Drosophila Research Center. Bloomington stock center (http://flybase.bio.indiana.edu/) provided all the Gal4 drivers utilized. *Drosophila* stocks and crosses were maintained on standard *Drosophila* medium at 25 °C, unless otherwise indicated. *Drosophila* locomotion activity was measured utilizing *Drosophila* Activity Monitor systems DAM2 (Trikinetics, Waltham, MA, USA) to record the number of climbing events of each fly in 45 min, banging the vials every 20 s. Climbing performance of each fly population (around 100 male flies 2–5 days after eclosion per genotype) was represented plotting the total number of climbing events of each fly population. Numbers corresponding to the climbing events of each fly of the population were ascending ordered and plotted as a curve. Negative geotaxis assay was performed according to the standard protocols.^27^

### DNA constructs

Full-length cDNA encoding human wild-type FUS (aa 1–526) was subcloned in pcDNA3.0 by PCR amplification of Flag FUS originally cloned in the pTRE2 vector.^28, 29^ FUSMM construct, carrying four C-terminal ALS-associated mutations (R521G, R522G, R524S, and P525L) was generated introducing the corresponding nucleotide substitutions in the reverse PCR primer and subcloning the mutated DNA in pcDNA3.0. Pur-alpha was PCR amplified from a rat cDNA library and cloned in pcDNA3.0-HA.^30^ HA-Pur-alpha, Flag-FUSWT, and Flag-FUSMM were subcloned in pUAST-attB vector to generate transgenic flies using the phiC31 integrase system. All constructs made by PCR were sequence verified.

### Antibodies

Sources of commercial antibodies were as follows: anti-FUS (Santa Cruz, Dallas, TX, USA; 4H11), anti-Pur-alpha (Abcam, Cambridge, UK; ab77734), anti-Pur-alpha (Abcam; ab79936), anti-HA (Santa Cruz; Y-11), anti-HA (Santa Cruz; F-7), anti-Flag (Sigma-Aldrich, St. Louis, MO, USA; M2 and M2 affinity gel), anti-Flag fluorescein isothiocyanate (FITC) conjugated (Sigma-Aldrich; M2), anti-Phospho-eIF2-alpha (Cell Signaling Technology, Beverly, MA, USA; D9G8), anti-eIF2-alpha (Cell Signaling Technology; D7D3), anti-cleaved caspase-3 (Cell Signaling Technology; Asp175), anti-NeuN (Merck Millipore, Billerica, MA, USA; A60), anti-TIAR (BD Biosciences, Erembodegem, Belgium), anti-GAPDH (Chemicon-Merck Millipore), anti-puromycin 3RH11 monoclonal antibody (Kerafast, Boston, MA, USA), anti-FUS (Abcam; ab84078), anti-Islet-1/2 (DSHB, Iowa City, IA, USA; 39.4D5). Mouse monoclonal antibody specific for ribosomal protein S19 were prepared in our laboratory.^31^ FITC, Rhodamine, and aminomethylcoumarin-conjugated affinity-purified secondary anti'bodies, selected for absent cross-reaction, were from Jackson ImmunoResearch (West Grove, PA, USA).

### Biochemical miscellaneous procedures

Tissue homogenization and GST pull-down experiments were carried out as previously described.^32^ Briefly, four mouse brains were harvested in 20 ml of HB (150 mM NaCl, 20 mM Tris (pH 7.5), 1 mM DTT, 1 mM EDTA, and protease inhibitors cocktail (Roche, Mannheim, Germany), homogenized, and the homogenate was spin at 3000 *g* for 5 min. Triton X-100 to the final concentration of 1% was added to the supernatant and the extract was incubate at 4 °C under constant rotation for 1 h. After centrifugation at 100 000 *g* for 45 min at 4 °C we collected the supernatant and measured protein concentration with BCA PROTEIN ASSAY KIT (Pierce-Thermo Fisher Scientific, Waltham, MA, USA). Twenty-five  milligrams of brain Triton X-100 extract were flowed 10 times through columns loaded with 500 *μ*g of each GST fusion bound to Glutathione Sepharose Fast Flow beads (GE Healthcare Life Sciences, Pittsburgh, PA, USA).

Immunoprecipitations were performed from cell lysates generated in lysis buffer (20 mM Tris (pH 7.5), 100 mM NaCl, 50 mM NaF, 1% Triton X-100) in the presence of protease inhibitor cocktail (Roche) and 1 mM orthovanadate. SDS-PAGE and western blotting were performed according to the standard protocols. Maldi-mass spectrometry was carried out by Telethon Proteomics Facility at the ISS (Istituto Superiore di Sanità), directed by Marco Crescenzi, as previously described.^33^ *In vitro* translated were prepared according to the manufacturer's protocols (TNT Quick Coupled Transcription/Translation Systems, Invitrogen-Thermo Fisher Scientific, Waltham, MA, USA).

To separate polyribosomes and ribosomal subunits, HEK293 cells were collected by scraping and then resuspended in lysis buffer (10 mM Tris-HCl (pH 7.5), 10 mM NaCl and 10 mM MgCl_2_, 1 mg/ml 0.5% NP-40, aprotinin, 1 mg/ml leupeptin, 1 mg/ml pepstatinA, and 100 mg/ml phenylmethanesulfonylfluoride). After incubation in ice for 20 min, the extract was centrifuged for 15 min in a microcentrifuge at a maximum speed at 4 °C and the supernatant (cytoplasmic extract) was loaded onto 10–30% linear sucrose gradient containing 30 mM Tris-HCl (pH 7.5), 100 mM NaCl and 10 mM MgCl_2_. Gradients were centrifuged in a Beckman SW 41 rotor for 4.5 h at 151 000* g* and collected in 11 fractions while monitoring the absorbance at 260 nm. The pellet was resuspended in the first fraction (polyribosomes). All fractions were precipitated with trichloroacetic acid, washed with acetone, dried, resuspended in loading buffer, and analyzed by western blot.

### Puromycin incorporation assay

HeLa cells were treated for 10 min with 10 *μ*g/ml of puromycin at 37 ºC. After two rapid washes in phosphate-buffered saline (PBS) cells were fixed with 4% paraformaldehyde in PBS. Blocking and antibody-staining (3RH11 antibody; Kerafast) were performed in PBS, 1% bovine serum albumin (BSA) buffer.

### Cell culture and immunofluorescence

HeLa and HEK293 cell lines were originally purchased from ATCC (Teddington, UK). NSC-34 cells were originally obtained from N. R. Cashman (University of Toronto, Toronto, ON, Canada). Transfection experiments were performed using Lipofectamine 2000 (Invitrogen-Thermo Fisher Scientific) according to the manufacturer's protocol. For immunofluorescence experiments cells were grown on glass coverslips and fixed in 4% paraformaldehyde in 60 mM phosphate buffer (pH 7.4). Mouse spinal cord sections were blocked and immunostained in BDB (BSA dilution buffer) containing 3% BSA, 0.3% Triton X-100, 20 mM phosphate buffer (pH 7.4), and 450 mM NaCl. Whole mount Drosophila brain were dissected and stained according to a standard protocol.^34^ Immunofluorescence analysis was carried out using Zeiss LSM 510 confocal microscopy, an Axioplan (Carl Zeiss, Jena, Germany) epi-fluorescent microscope equipped with CCD camera (Photometrics, Tucson, AZ, USA) and a Leica SP2 confocal microscope. Fluorescence images were processed using Adobe Photoshop.

### Generation and maintenance of human iPSCs

Skin biopsies of informed donor ALS patients were used to generate dermal fibroblasts cultured in fibroblast basal medium (Lonza, Basel, Switzerland) containing 15% feta bovine serum (FBS), 1 × l-Glu, 1 × penicillin–streptomycin (all from Sigma-Aldrich). ALS and control fibroblasts were infected in serum-free conditions and in the presence of 4 mg/ml polybrene with the lentiviral vector hSTEMCCA,^35^ which carries the four reprogramming factors OCT4, SOX2, KLF4, and cMYC in a single polycistronic unit. Seven days after infection fibroblasts were seeded on a feeder layer of mitomycin C-treated primary mouse embryonic fibroblasts (PMEF-CF; Millipore) and the next day the medium was changed in HUESM (DMEM-F12+Glutamax, Life Technologies-Thermo Fisher Scientific, Waltham, MA, USA; 20% knockout serum replacement, Life Technologies-Thermo Fisher Scientific; 1 × non-essential aminoacids, NEAA, Life Technologies-Thermo Fisher Scientific; 1 × penicillin–streptomycin, 0.1 mM *β*-mercaptoethanol, Gibco-Thermo Fisher Scientific, Waltham, MA, USA) supplemented with basic fibroblast growth factor (10 ng/ml; BD Biosciences). Twenty days after infection the medium was replaced with Nutristem-XF (Biological Industries, Beit-Haemek, Israel). iPSC colonies were manually picked, fragmented, and plated on PMEF-CF coated wells. Established iPSC lines were maintained in Nutristem-XF on hESC-qualified Matrigel-coated plates (BD Biosciences) and passaged every 4–5 days with 1 mg/ml Dispase (Gibco-Thermo Fisher Scientific).

### Generation of iPSCs carrying the FUS-P525L mutation by transcription activator-like effector nucleases

Using a genome editing approach based on transcription activator-like effector nucleases (TALENs) we introduced the P525L mutation in the endogenous FUS locus of an iPSC-WT line. The TALE-TN software (available at https://tale-nt.cac.cornell.edu) provided the sequence of a TALEN pair specific for exon 15 in the FUS locus. We generated a homology directed repair (HDR) donor construct including the P525L mutation and a selection cassette containing an independent promoter (PGK) that drives the expression of the PUΔTK bifunctional protein,^36^ which in turn confers resistance to puromycin and sensitivity to ganciclovir (GCV). After co-transfection of this construct with the FUS C-term TALENs, we isolated individual puromycin-resistant clones. HDR stimulated by TALENs promoted the insertion of the selection cassette flanked by the enhanced piggyBac (ePB) terminal repeats. First we selected clones containing the cassette in both FUS alleles, which represented a homozygous mutant. We transfected such cells with a modified PB transposase, which was competent for excision but not for the re-integration of the cassette. iPSCs retaining the exogenous cassette were counter-selected by GCV. Finally we isolated individual GCV-resistant clones and the presence of the homozygous P525L mutation was confirmed by sequencing.

### Differentiation of iPSCs into neural cells

iPSCs around passage 10–20 were plated in HUESM supplemented with SMAD inhibitors (SB/DM; 10 *μ*M SB431542 and 2.5 *μ*M Dorsomorphin, both from Miltenyi Biotec, Bologna, Italy) and were considered as day 0 of differentiation (D0). From D4, in the presence of SB/DM, the medium was gradually replaced with N2M (DMEM-F12; 1 × N2 Supplement, Life Technologies-Thermo Fisher Scientific; 1 × NEAA, Life Technologies-Thermo Fisher Scientific; 2 *μ*g/ml heparin, Sigma-Aldrich). We started at D4 with 75% HUESM and 25% N2M that reached 25% HUESM and 75% N2M at D8. From D10 to D14, differentiating neural progenitors were cultured in N2M supplemented with 0.1 *μ*M all-*trans* retinoic acid (RA; Sigma-Aldrich). At D14 neural rosettes were manually detached to generate floating neurospheres, maintained in N2M supplemented with 0.1 *μ*M RA and 1 *μ*M purmorphamine (sc-202705; Santa Cruz). At D28 neurospheres were plated in poly-l-lysine (Sigma-Aldrich) and Natural Mouse Laminin (Invitrogen-Thermo Fisher Scientific) coated glass coverslips for immunostaining, in N2M supplemented with 10% FBS. The day after, the medium was replaced with N2M supplemented with 10 ng/ml brain-derived neurotrophic factor, 10 ng/ml glial cell line-derived neurotrophic factor, and 10 ng/ml insulin-like growth factor (all from PreproTech, London, UK), 1 *μ*M cAMP and 200 ng/ml l-ascorbic acid (both from Sigma-Aldrich), 0.05 *μ*M RA and 0.5 *μ*M purmorphamine. Motoneuronal cells were fixed for immunostaining at day 34.

## Abbreviations

ALS: amyotrophic lateral sclerosis
BSA: bovine serum albumin
DMEM: Dulbecco's modified Eagle's medium
eIF2-alpha: eukaryotic translation initiation factor 2 alpha
ELAV: embryonic lethal abnormal vision
FBS: fetal bovine serum
FITC: fluorescein isothiocyanate
FUS: Fused in sarcoma
Gal4: galactose-responsive transcription factor
GST: glutathione *S*-transferase
HA: hemagglutin
iPSCs: induced pluripotent stem cells
NonA: no on or off transient A
PABP: poly(A) binding protein
PBS: phosphate-buffered saline
NY-NLS: proline/tyrosine-nuclear localization signal
PY-NLS: proline/tyrosine-nuclear localization signal
Pur-alpha: purine-rich binding protein-alpha
RAR-alpha: retinoic acid receptor alpha
SGs: stress granules
TIAR: TIA-1 related protein
TDP-43: TAR DNA-binding protein-43

## Supplementary Material

Supplementary Informations

## References

[bib1] 1Renton AE, Chio A, Traynor BJ. State of play in amyotrophic lateral sclerosis genetics. Nat Neurosci 2014; 17: 17–23. 24369373 10.1038/nn.3584 PMC4544832

[bib2] 2Vance C, Rogelj B, Hortobagyi T, De Vos KJ, Nishimura AL, Sreedharan J et al. Mutations in FUS, an RNA processing protein, cause familial amyotrophic lateral sclerosis type 6. Science 2009; 323: 1208–1211. 19251628 10.1126/science.1165942 PMC4516382

[bib3] 3Kwiatkowski TJ Jr, Bosco DA, Leclerc AL, Tamrazian E, Vanderburg CR, Russ C et al. Mutations in the FUS/TLS gene on chromosome 16 cause familial amyotrophic lateral sclerosis. Science 2009; 323: 1205–1208. 19251627 10.1126/science.1166066

[bib4] 4Da Cruz S, Cleveland DW. Understanding the role of TDP-43 and FUS/TLS in ALS and beyond. Curr Opin Neurobiol 2011; 21: 904–919. 21813273 10.1016/j.conb.2011.05.029 PMC3228892

[bib5] 5Zhang ZC, Chook YM. Structural and energetic basis of ALS-causing mutations in the atypical proline-tyrosine nuclear localization signal of the Fused in Sarcoma protein (FUS). Proc Natl Acad Sci USA 2012; 109: 12017–12021. 22778397 10.1073/pnas.1207247109 PMC3409756

[bib6] 6Dormann D, Rodde R, Edbauer D, Bentmann E, Fischer I, Hruscha A et al. ALS-associated fused in sarcoma (FUS) mutations disrupt Transportin-mediated nuclear import. EMBO J 2010; 29: 2841–2857. 20606625 10.1038/emboj.2010.143 PMC2924641

[bib7] 7Graebsch A, Roche S, Niessing D. X-ray structure of Pur-alpha reveals a Whirly-like fold and an unusual nucleic-acid binding surface. Proc Natl Acad Sci USA 2009; 106: 18521–18526. 19846792 10.1073/pnas.0907990106 PMC2765457

[bib8] 8Kanai Y, Dohmae N, Hirokawa N. Kinesin transports RNA: isolation and characterization of an RNA-transporting granule. Neuron 2004; 43: 513–525. 15312650 10.1016/j.neuron.2004.07.022

[bib9] 9White MK, Johnson EM, Khalili K. Multiple roles for Puralpha in cellular and viral regulation. Cell Cycle 2009; 8: 1–7. 19182532 10.4161/cc.8.3.7585 PMC2683411

[bib10] 10Darbinian N, Cui J, Basile A, Del Valle L, Otte J, Miklossy J et al. Negative regulation of AbetaPP gene expression by pur-alpha. J Alzheimers Dis 2008; 15: 71–82. 18780968 10.3233/jad-2008-15106

[bib11] 11Johnson EM, Kinoshita Y, Weinreb DB, Wortman MJ, Simon R, Khalili K et al. Role of Pur alpha in targeting mRNA to sites of translation in hippocampal neuronal dendrites. J Neurosci Res 2006; 83: 929–943. 16511857 10.1002/jnr.20806

[bib12] 12Hokkanen S, Feldmann HM, Ding H, Jung CK, Bojarski L, Renner-Muller I et al. Lack of Pur-alpha alters postnatal brain development and causes megalencephaly. Hum Mol Genet 2012; 21: 473–484. 22010047 10.1093/hmg/ddr476

[bib13] 13Sareen D, O'Rourke JG, Meera P, Muhammad AK, Grant S, Simpkinson M et al. Targeting RNA foci in iPSC-derived motor neurons from ALS patients with a C9ORF72 repeat expansion. Sci Transl Med 2013; 5:208ra149. 10.1126/scitranslmed.3007529 PMC4090945 24154603

[bib14] 14Xu Z, Poidevin M, Li X, Li Y, Shu L, Nelson DL et al. Expanded GGGGCC repeat RNA associated with amyotrophic lateral sclerosis and frontotemporal dementia causes neurodegeneration. Proc Natl Acad Sci USA 2013; 110: 7778–7783. 23553836 10.1073/pnas.1219643110 PMC3651485

[bib15] 15Shelkovnikova TA, Robinson HK, Troakes C, Ninkina N, Buchman VL. Compromised paraspeckle formation as a pathogenic factor in FUSopathies. Hum Mol Genet 2014; 23: 2298–2312. 24334610 10.1093/hmg/ddt622 PMC3976330

[bib16] 16Bilican B, Serio A, Barmada SJ, Nishimura AL, Sullivan GJ, Carrasco M et al. Mutant induced pluripotent stem cell lines recapitulate aspects of TDP-43 proteinopathies and reveal cell-specific vulnerability. Proc Natl Acad Sci USA 2012; 109: 5803–5808. 22451909 10.1073/pnas.1202922109 PMC3326463

[bib17] 17Kedersha NL, Gupta M, Li W, Miller I, Anderson P. RNA-binding proteins TIA-1 and TIAR link the phosphorylation of eIF-2 alpha to the assembly of mammalian stress granules. J Cell Biol 1999; 147: 1431–1442. 10613902 10.1083/jcb.147.7.1431 PMC2174242

[bib18] 18Kedersha N, Cho MR, Li W, Yacono PW, Chen S, Gilks N et al. Dynamic shuttling of TIA-1 accompanies the recruitment of mRNA to mammalian stress granules. J Cell Biol 2000; 151: 1257–1268. 11121440 10.1083/jcb.151.6.1257 PMC2190599

[bib19] 19Schmidt EK, Clavarino G, Ceppi M, Pierre P. SUnSET, a nonradioactive method to monitor protein synthesis. Nat Methods 2009; 6: 275–277. 19305406 10.1038/nmeth.1314

[bib20] 20David A, Dolan BP, Hickman HD, Knowlton JJ, Clavarino G, Pierre P et al. Nuclear translation visualized by ribosome-bound nascent chain puromycylation. J Cell Biol 2012; 197: 45–57. 22472439 10.1083/jcb.201112145 PMC3317795

[bib21] 21Chen N, Onisko B, Napoli JL. The nuclear transcription factor RARalpha associates with neuronal RNA granules and suppresses translation. J Biol Chem 2008; 283: 20841–20847. 18495661 10.1074/jbc.M802314200 PMC2475717

[bib22] 22Gallia GL, Darbinian N, Jaffe N, Khalili K. Single-stranded nucleic acid-binding protein, Pur alpha, interacts with RNA homologous to 18 S ribosomal RNA and inhibits translation *in vitro*. J Cell Biochem 2001; 83: 355–363. 11596104 10.1002/jcb.1247

[bib23] 23Kim HJ, Raphael AR, Ladow ES, McGurk L, Weber RA, Trojanowski JQ et al. Therapeutic modulation of eIF2alpha phosphorylation rescues TDP-43 toxicity in amyotrophic lateral sclerosis disease models. Nat Genet 2014; 46: 152–160. 24336168 10.1038/ng.2853 PMC3934366

[bib24] 24Mitchell JC, McGoldrick P, Vance C, Hortobagyi T, Sreedharan J, Rogelj B et al. Overexpression of human wild-type FUS causes progressive motor neuron degeneration in an age- and dose-dependent fashion. Acta Neuropathol 2013; 125: 273–288. 22961620 10.1007/s00401-012-1043-z PMC3549237

[bib25] 25Sabatelli M, Moncada A, Conte A, Lattante S, Marangi G, Luigetti M et al. Mutations in the 3' untranslated region of FUS causing FUS overexpression are associated with amyotrophic lateral sclerosis. Hum Mol Genet 2013; 22: 4748–4755. 23847048 10.1093/hmg/ddt328

[bib26] 26Xia R, Liu Y, Yang L, Gal J, Zhu H, Jia J. Motor neuron apoptosis and neuromuscular junction perturbation are prominent features in a Drosophila model of Fus-mediated ALS. Mol Neurodegener 2012; 7: 10. 22443542 10.1186/1750-1326-7-10 PMC3325858

[bib27] 27Nichols CD, Becnel J, Pandey UB. Methods to assay Drosophila behavior. J Vis Exp 2012; 61: 3791–3795. 10.3791/3795 PMC3671839 22433384

[bib28] 28Cozzolino M, Pesaresi MG, Gerbino V, Grosskreutz J, Carri MT. Amyotrophic lateral sclerosis: new insights into underlying molecular mechanisms and opportunities for therapeutic intervention. Antioxid Redox Signal 2012; 17: 1277–1330. 22413952 10.1089/ars.2011.4328

[bib29] 29Gerbino V, Carri MT, Cozzolino M, Achsel T, Mislocalised FUS. mutants stall spliceosomal snRNPs in the cytoplasm. Neurobiol Dis 2013; 55: 120–128. 23523636 10.1016/j.nbd.2013.03.003

[bib30] 30Cestra G, Toomre D, Chang S, De Camilli P. The Abl/Arg substrate ArgBP2/nArgBP2 coordinates the function of multiple regulatory mechanisms converging on the actin cytoskeleton. Proc Natl Acad Sci USA 2005; 102: 1731–1736. 15659545 10.1073/pnas.0409376102 PMC547834

[bib31] 31Chiocchetti A, Gibello L, Carando A, Aspesi A, Secco P, Garelli E et al. Interactions between RPS19, mutated in Diamond-Blackfan anemia, and the PIM-1 oncoprotein. Haematologica 2005; 90: 1453–1462. 16266891

[bib32] 32Slepnev VI, Ochoa GC, Butler MH, Grabs D, De Camilli P. Role of phosphorylation in regulation of the assembly of endocytic coat complexes. Science 1998; 281: 821–824. 9694653 10.1126/science.281.5378.821

[bib33] 33Lalle M, Camerini S, Cecchetti S, Sayadi A, Crescenzi M, Pozio E. Interaction network of the 14-3-3 protein in the ancient protozoan parasite Giardia duodenalis. J Proteome Res 2012; 11: 2666–2683. 22452640 10.1021/pr3000199

[bib34] 34Wu JS, Luo L. A protocol for dissecting *Drosophila melanogaster* brains for live imaging or immunostaining. Nat Protoc 2006; 1: 2110–2115. 17487202 10.1038/nprot.2006.336

[bib35] 35Somers A, Jean JC, Sommer CA, Omari A, Ford CC, Mills JA et al. Generation of transgene-free lung disease-specific human induced pluripotent stem cells using a single excisable lentiviral stem cell cassette. Stem Cells 2010; 28: 1728–1740. 20715179 10.1002/stem.495 PMC3422663

[bib36] 36Chen YT, Bradley A. A new positive/negative selectable marker, puDeltatk, for use in embryonic stem cells. Genesis 2000; 28: 31–35. 11020714 10.1002/1526-968x(200009)28:1<31::aid-gene40>3.0.co;2-k

