# Supplementary figures and images for: Pur-alpha functionally interacts with FUS carrying ALS-associated mutations
Source: Cell Death Dis. 2015 Oct 22;6(10):e1943–. doi: 10.1038/cddis.2015.295 (PMC4632316; doi:10.1038/cddis.2015.295)

## Slide 1
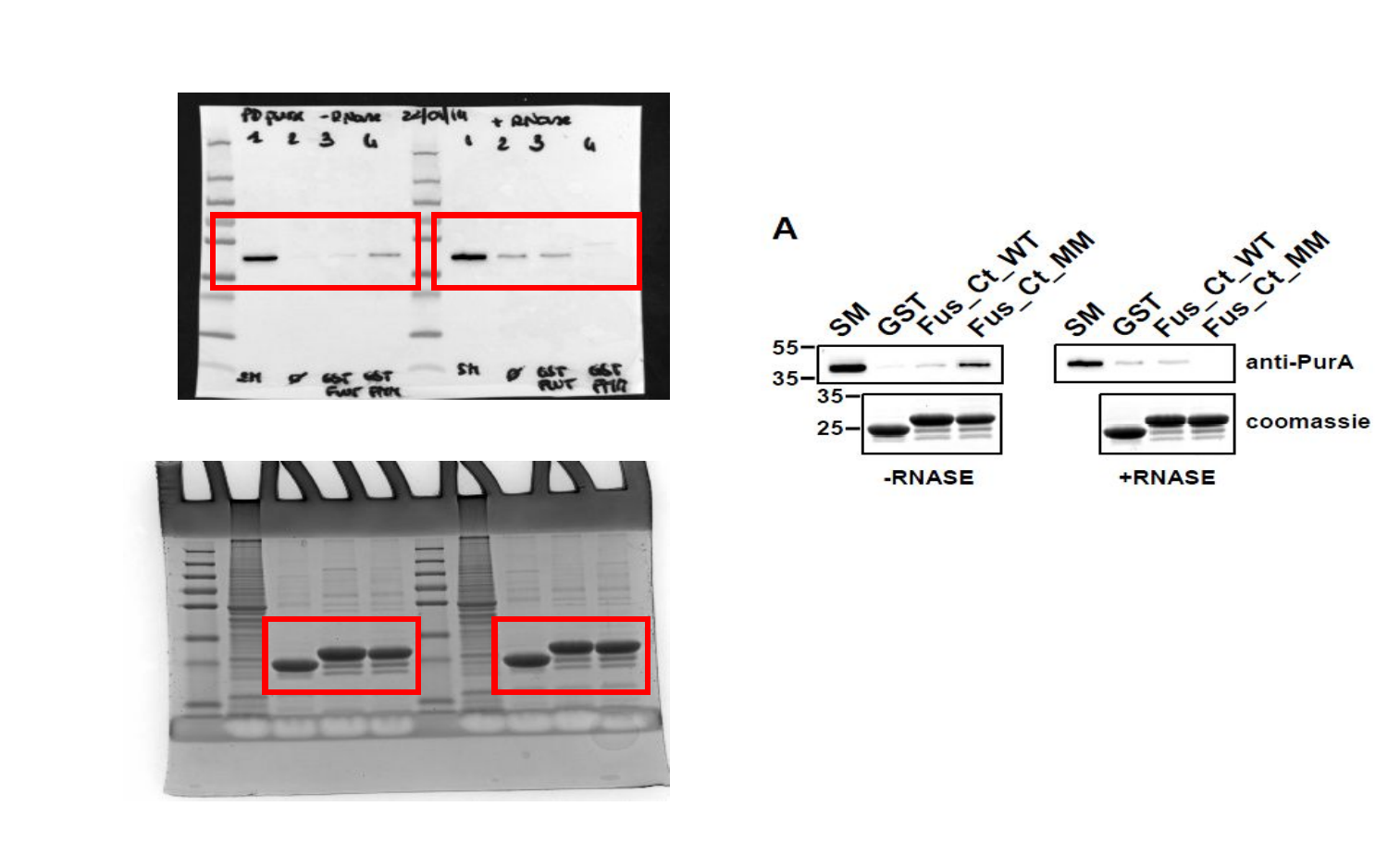

## Slide 2
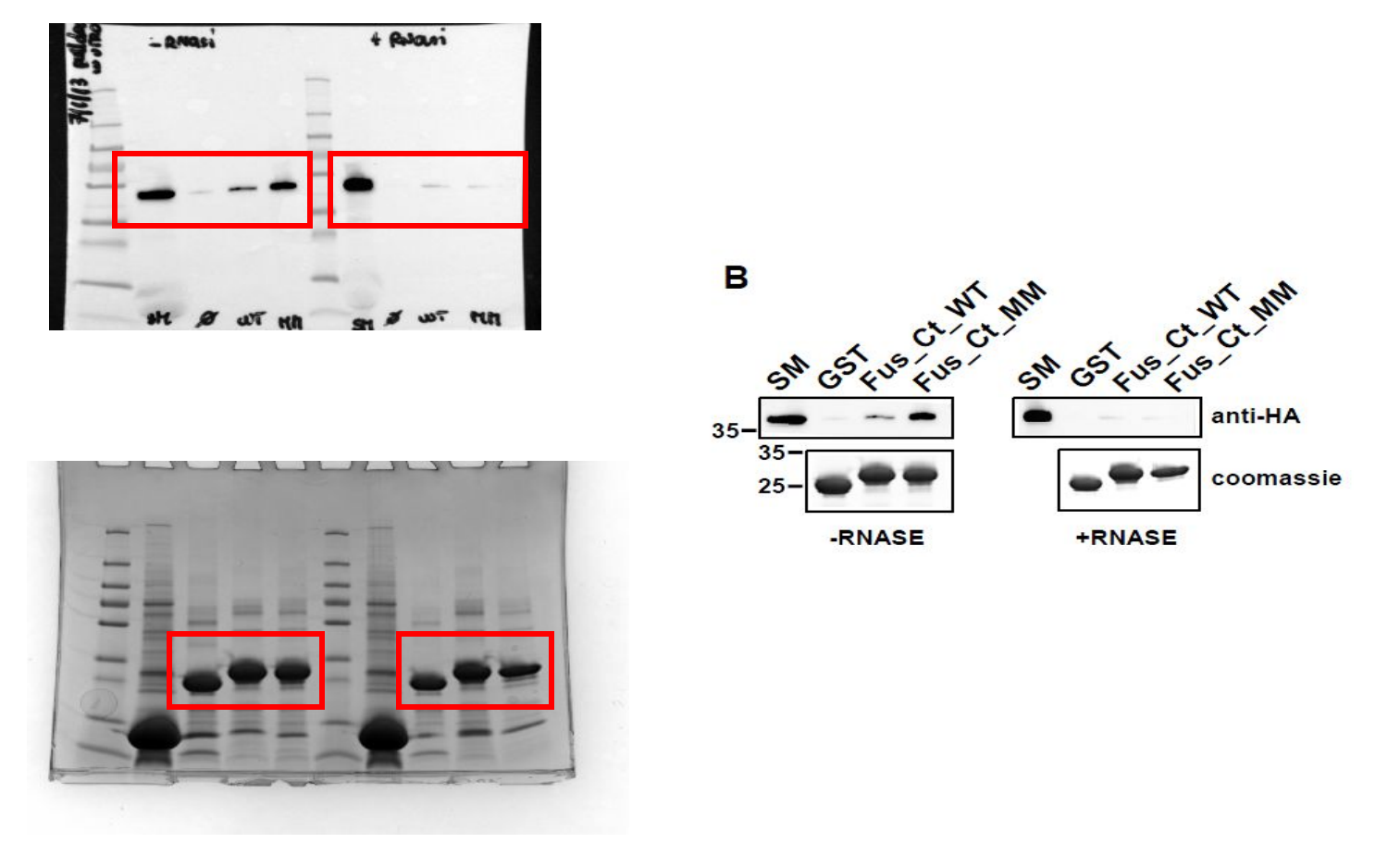

## Slide 3
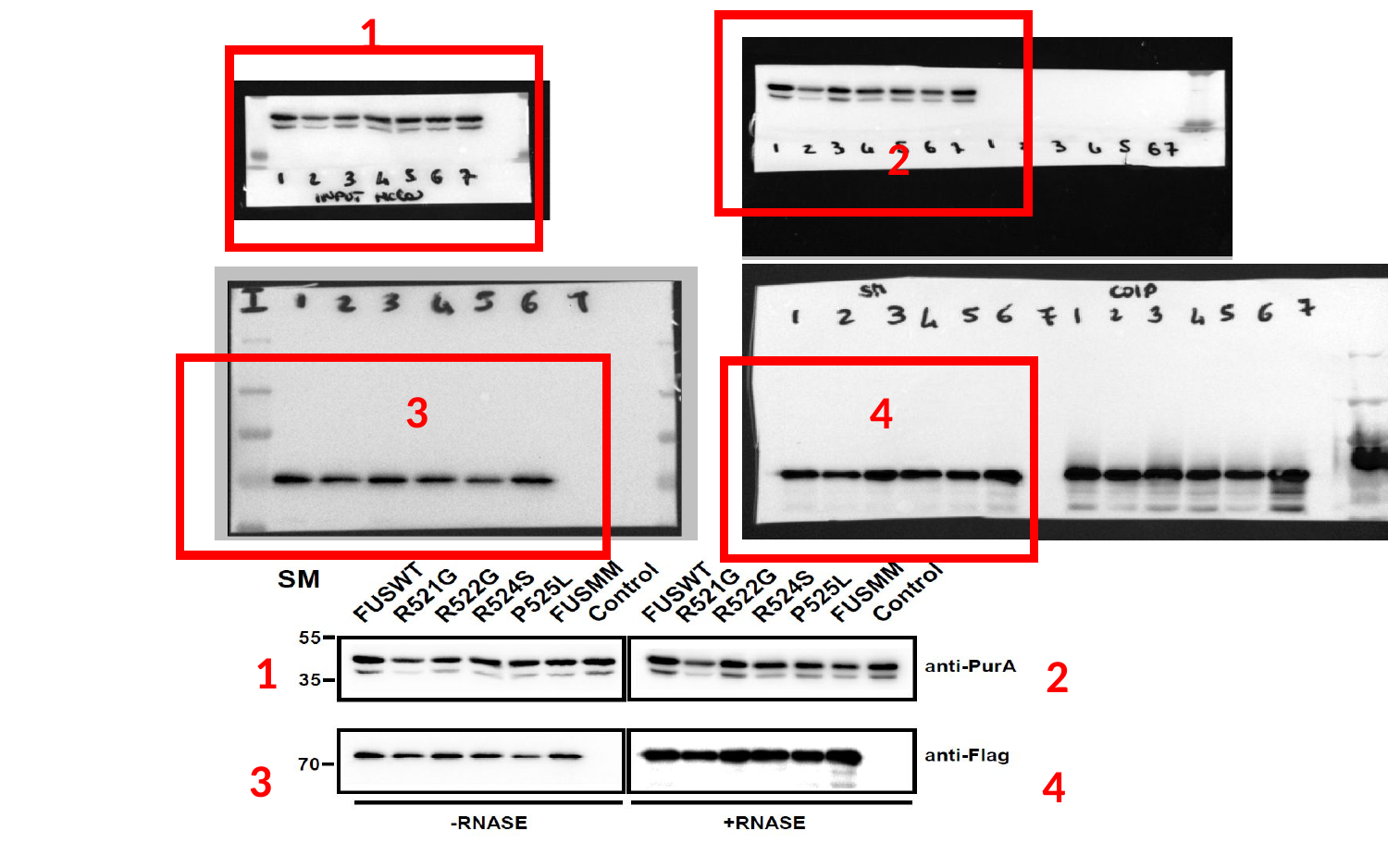

1
2
3
4
1
2
3
4

## Slide 4
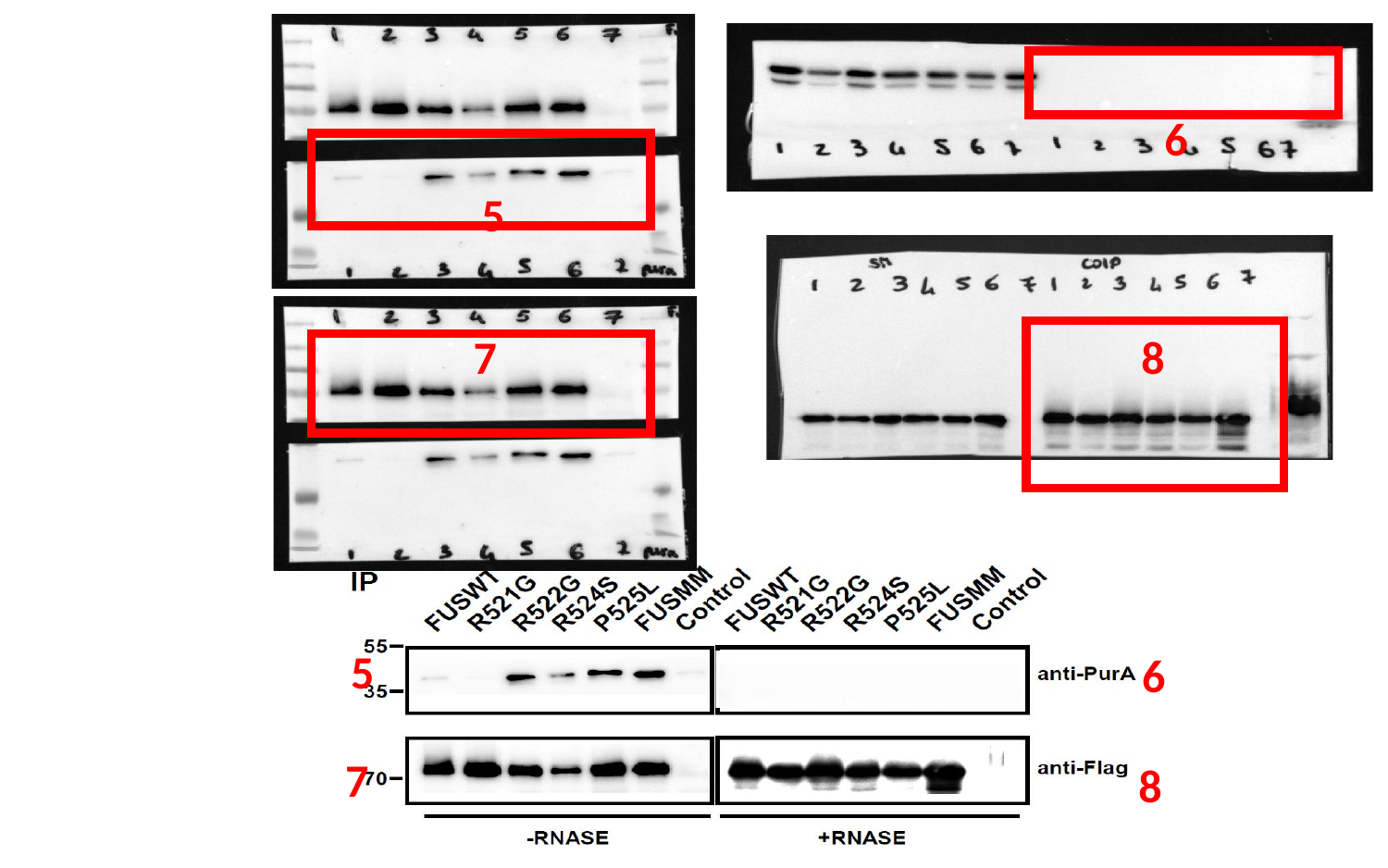

6
6
5
7
8
7
8
5
6
7
8

## Slide 5
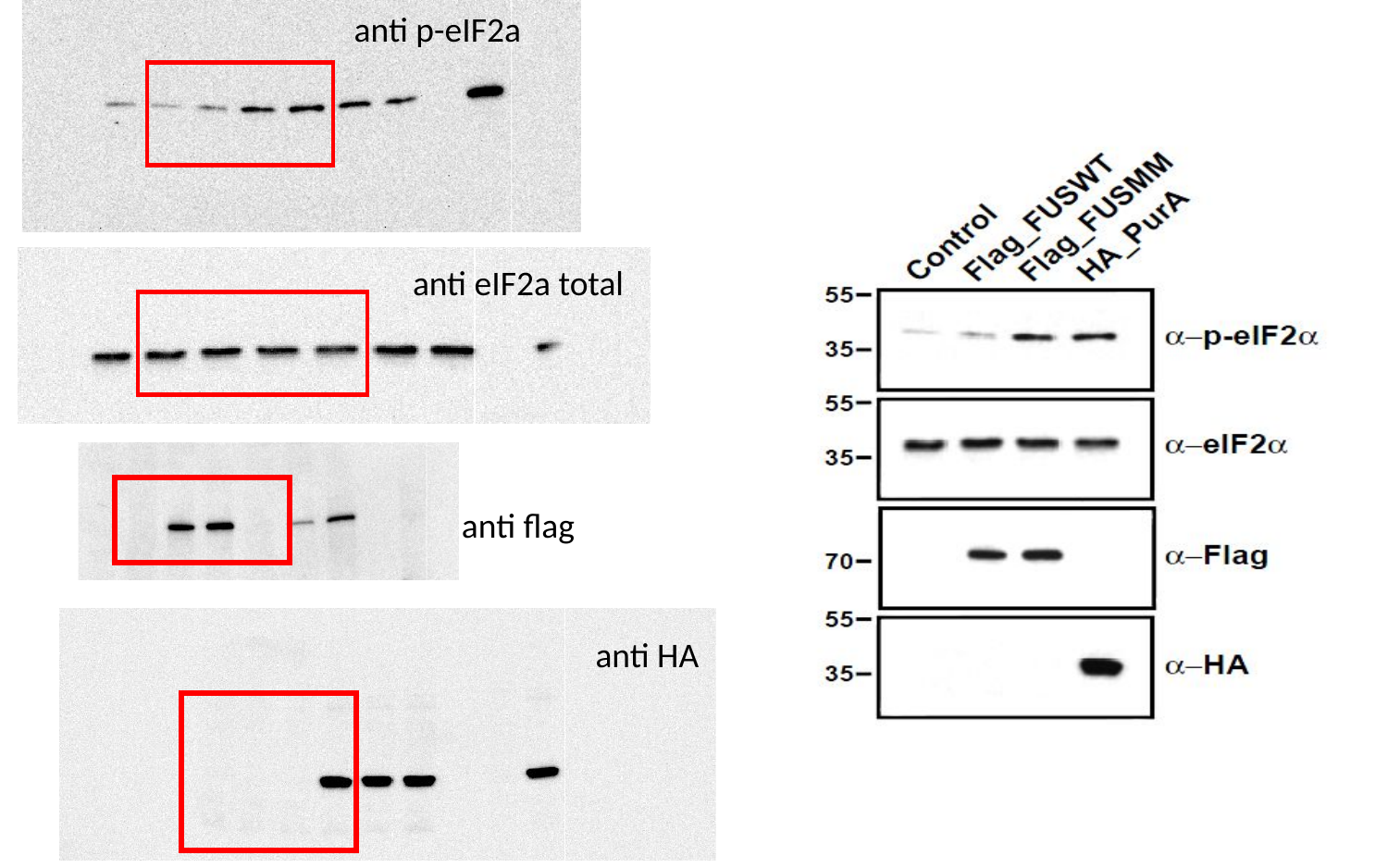

anti p-eIF2a
anti eIF2a total
anti flag
anti HA

## Slide 6
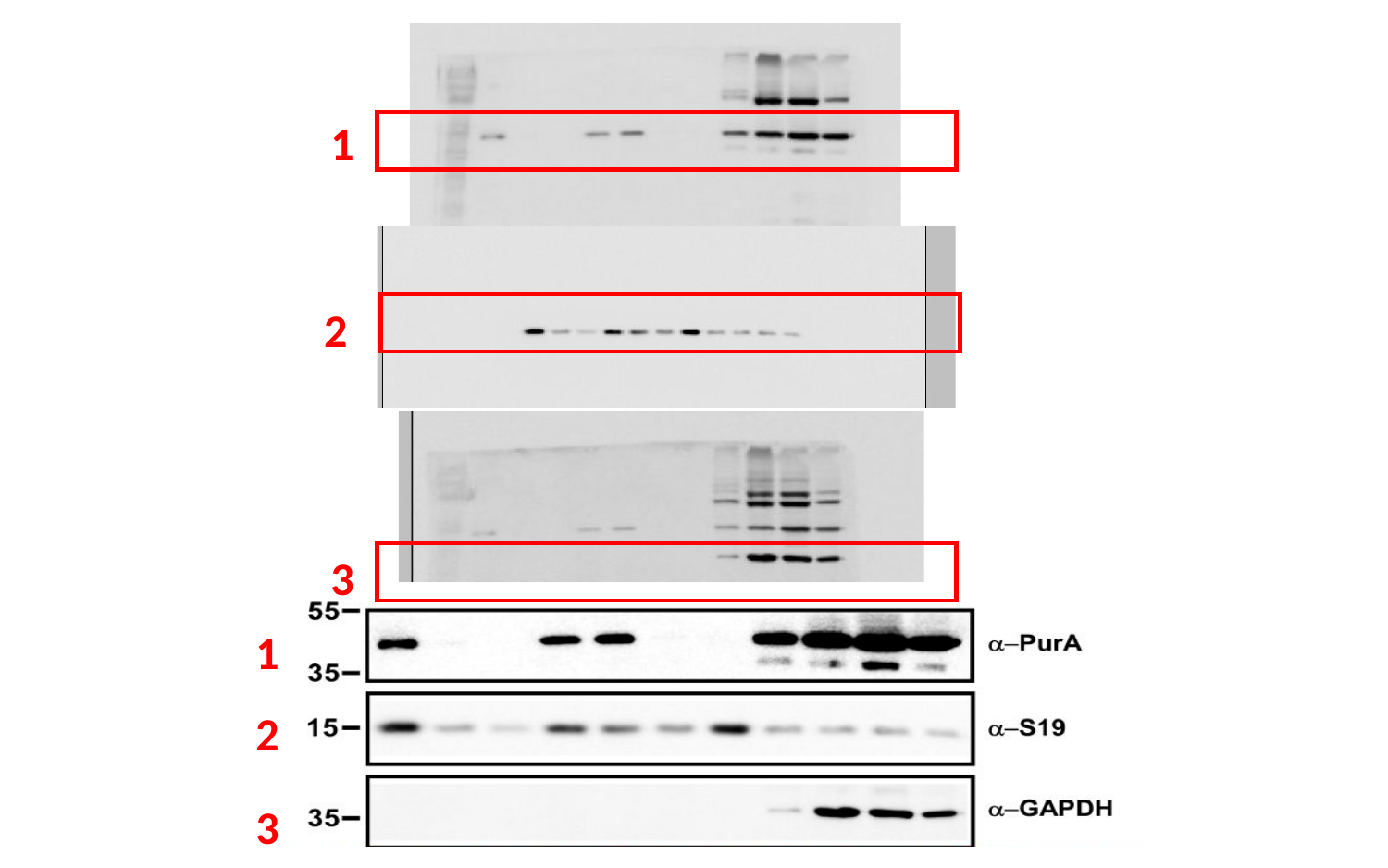

1
2
3
1
2
3

## Slide 7
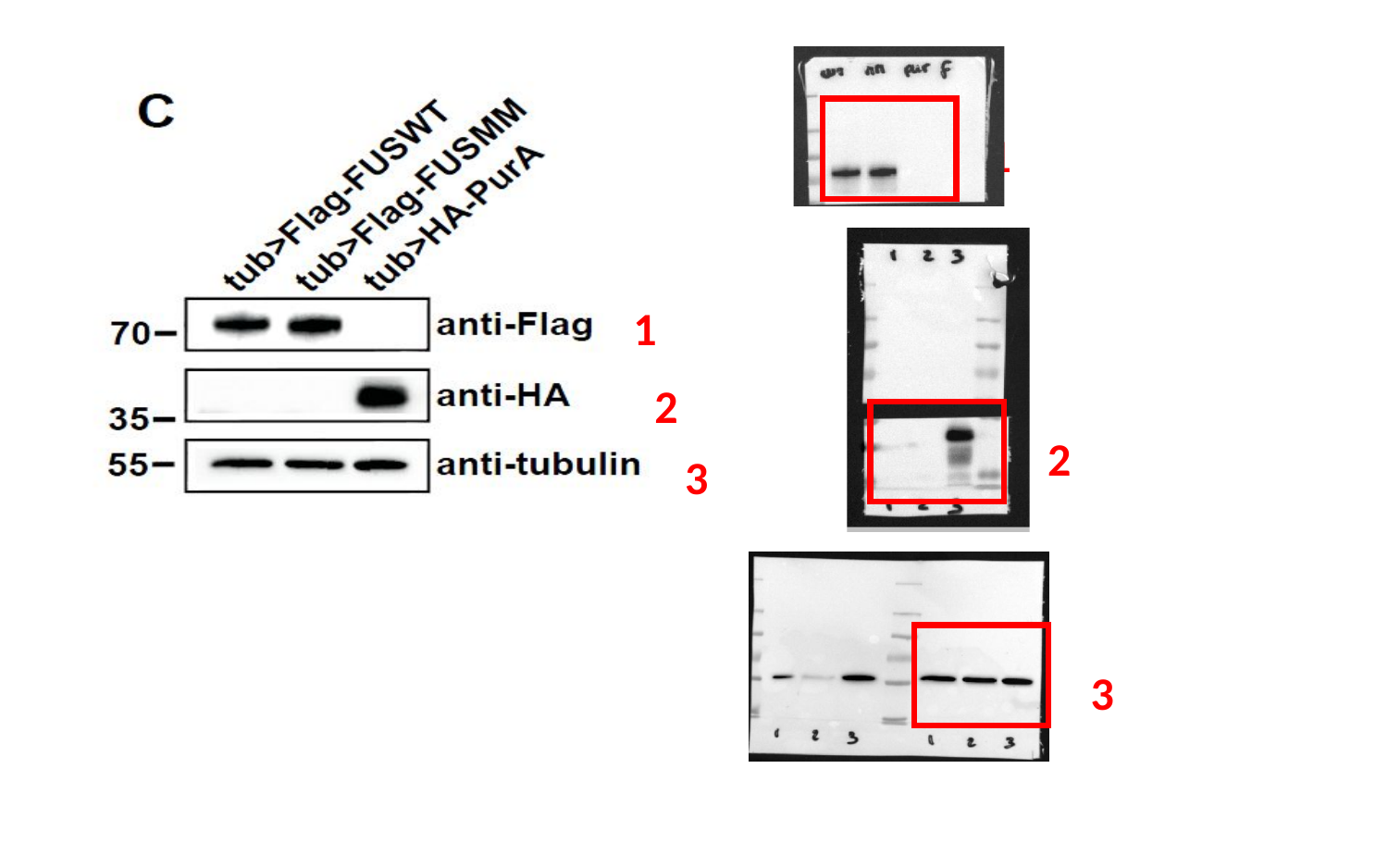

1
1
2
2
3
3

Supplement: Supplementary Informations [file cddis2015295x1.ppt]
